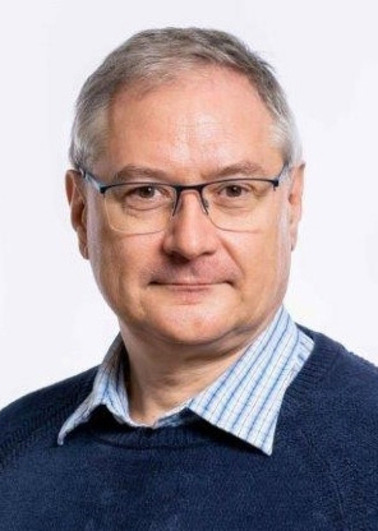# Electrochemical Hydrodimerization of Lignocellulose‐Derived Carbonyls in Aqueous Electrolytes for Biobased Polymer and Long‐chained Synfuel Production: A Review

**DOI:** 10.1002/cssc.202400638

**Published:** 2024-11-08

**Authors:** Robin Kunkel, Volkmar M. Schmidt

**Affiliations:** ^1^ Fraunhofer Institute for Chemical Technology ICT Department of Applied Electrochemistry Joseph-von-Fraunhofer-Str. 7 D-76327 Pfinztal Germany; ^2^ Mannheim University of Applied Sciences Institute of Chemical Process Engineering Paul-Wittsack-Str. 10 D-68163 Mannheim Germany

**Keywords:** Electrosynthesis, Furfural, Lignocellulose, Vanillin, 5-HMF

## Abstract

The transformation from fossil resources, crude oil and natural gas to biomass‐derived feedstocks is an urgent and major challenge for the chemical industry. The valorization of lignocellulose as renewable resource is a promising pathway offering access to a wide range of platform chemicals, such as vanillin, furfural and 5‐HMF. The subsequent conversion of such platform chemicals is one crucial step in the value‐added chain. The electrochemical hydrodimerization (EHD) is a sustainable tool for C−C coupling of these chemicals to their corresponding hydrodimers hydrovanilloin, hydrofuroin and 5,5′‐bis(hydroxymethyl)hydrofuroin (BHH). This review covers the current state of art concerning the mechanism of the electrochemical reduction of biobased aldehydes and studies targeting the electrochemical production of these hydrodimers in aqueous media. Moreover, the subsequent conversion of these hydrodimers to valuable additives, polymers and long carbon chain synfuels will be summarized offering a broad scope for their application in the chemical industry.

## Graphical abstract

**Electrochemistry meets biomass**: Lignocellulose‐derived carbonyls, such as vanillin, furfural and 5‐HMF, are versatile and green building blocks. Electrochemical hydrodimerization of these intermediates offers a sustainable pathway to long‐chained multifunctional precursors for biobased polymers and synfuels for heavy‐duty applications. This review covers mechanistic insights and current studies on the electrochemical hydrodimerization of these compounds and their subsequent chemical upgrading for application.

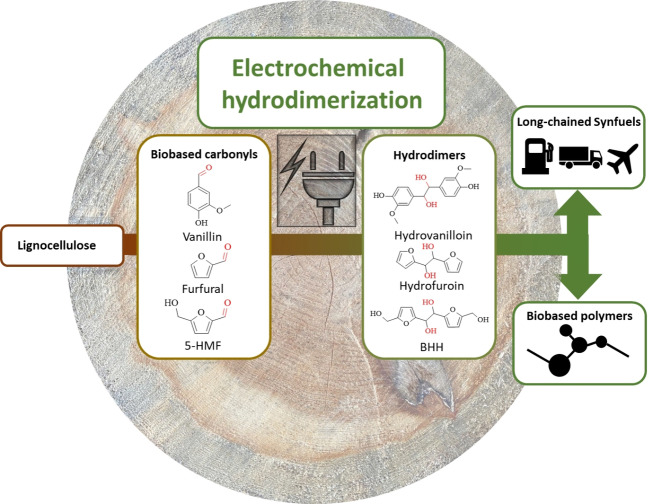

## Introduction

The valorization of biomass for the replacement of fossil‐based products is a global burning issue targeting many challenges of modern society, such as fossil fuel depletion, greenhouse gas (GHG) emissions, increasing energy demand or security of supply.[^1^, ^2^] Lignocellulose exhibits the highest potential as renewable feedstock for establishing a sustainable circular economy due to its abundance, low price and accessibility.^[3]^ The annual worldwide production of lignocellulose is 170 billion metric tons of which only 5 % are utilized by human. Lignocellulose consists of the three primary ingredients cellulose, hemicellulose and lignin, which can be depolymerized in several biobased platform chemicals.^[4]^ Analogously to the oil refineries the biobased platform chemicals can be converted in downstream processes to marketable products, such as (fine−)chemicals, biofuels or biobased polymers.^[3]^

To be competitive with existing fossil‐based production biobased processes need to be energy‐efficient, cost‐efficient and sustainable to meet the goals of a market‐driven Bioeconomy.^[3]^ The conversion of biobased platform chemicals *via* electrochemical processes lead to a total green value chain, as electrochemical conversions align with the principles of green chemistry, e. g. only electrons serve as reactants, toxic reagents can be avoided leading to high atom efficiencies. Furthermore, electrochemical processes can be operated under mild reaction conditions with respect to pressure and temperature.[^5^, ^6^] Moreover, decreasing electricity costs down to 0.02 € kWh^−1^ due to technical advancements in photovoltaic make electrosynthetic processes interesting for the production of chemicals on an industrial scale.^[7]^

Electrochemical hydrodimerization (EHD) can be described as a combination of electroreduction and C−C‐coupling of aromatic carbonyl‐ containing compounds or of hydrocarbons with C−C double‐bonds having electronegative functional groups attached. EHD is a powerful tool for carbon chain elongation. For example, the most known electrochemical process on an industrial scale is the Monsanto process with an annual production of 300,000 tons, which is the EHD of acrylonitrile to adiponitrile.^[6]^

**Figure 1:**
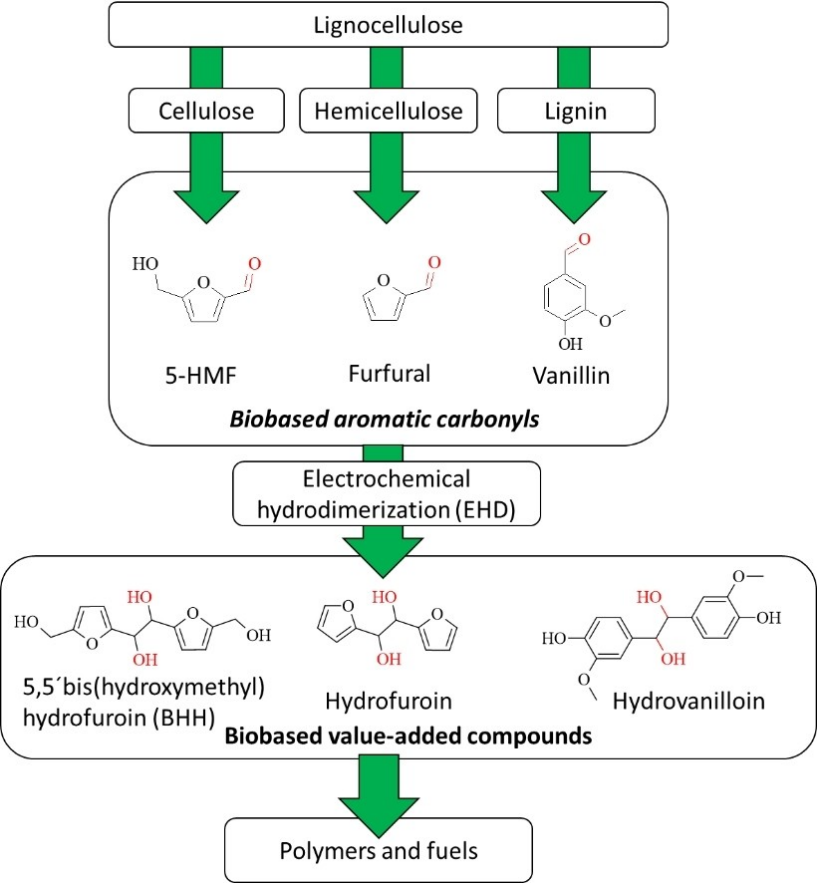
Value‐added chain for the production of renewable polymer and fuels from lignocellulose via biobased aromatic carbonyls and their corresponding hydrodimers obtained by EHD.

Similarly, EHD can be applied on to aromatic carbonyl‐containing compounds leading to their corresponding pinacols. Several aromatic biobased carbonyls are accessible from lignocellulosic biomass in well‐established processes, such as furfural, 5‐(hydroxy)methylfurfural (5‐HMF) and vanillin, which then can be converted to their pinacols hydrofuroin, 5,5′‐bis(hydroxymethyl)hydrofuroin (BHH) and hydrovanilloin (Figure 1).

In detail, the global furfural production from biomass is 300,000–700,000 tons per year, whereby China is the major contributor.[^8^, ^9^] The market price of furfural is ≈1,5 € kg^−1^ furfural.^[10]^ Furfural is obtained from pentosans, C5 sugars in the hemicellulose fraction in the lignocellulose, which account up to 35 % of the total biomass depending on their origin/sort.^[11]^ The conventional process is a pre‐treatment of the lignocellulosic biomass with sulfuric acid followed by hydrolysis with hot steam and lastly, an azeotropic distillation for refining is conducted.^[12]^ 5‐HMF is produced *via* dehydration of C6 sugars, fructose and glucose, which are obtained by depolymerization of cellulose. However, no large‐scale production is available by now due to process‐related challenges, such as formation of side products, difficulties in downstream separation and catalyst regeneration.^[13]^ This leads to a very high price of ≈6000 € kg^−1^ (Merck) for commercially available 5‐HMF. Currently, the largest scale production site of commercial 5‐HMF is operated by AVA Biochem since 2014 with an annual production of 300 tons per year.^[13]^ Both, furfural and 5‐HMF, are proposed as one of the top biobased intermediates from lignocellulosic biomass and, therefore, are expected to play an important role in the product portfolio of the biorefinery of the future.^[14]^

Biobased vanillin contributes to ≈15 % of the worldwide total vanillin production, which was in recent years 24,000–37,000 tons per year.[^15^, ^16^] Whereas the main fraction of the worldwide vanillin production is obtained from fossil fuels *via* the well‐established Rhodia process (Solvay), biobased vanillin is mainly obtained from the selective depolymerization of lignin, which is considered as side product in the paper and pulp industry.[^17^, ^18^] Borregaard, a Norwegian company, operates the biobased vanillin production from lignosulfonates on an industrial scale since over 50 years. The process is based on a thermo‐catalytic depolymerization in the presence of copper‐based catalysts and oxygen.^[15]^ More recently, innovative and more sustainable electrooxidation pathways for the depolymerization of lignins are investigated showing promising results with high vanillin selectivity and yields up to 7 wt.–%.^[19]^ Also other phenolic aromatic carbonyls are obtainable depending on the origin of the lignins, such as syringaldehyde, 4‐hydroxybenzaldehyde.^[20]^ However, the focus of research for subsequent use in the value‐chain is currently mostly laid on vanillin. For the sake of completeness, the production from rice bran oil should be mentioned, which, however, only contributes to very small parts of the worldwide vanillin production. The market price of biobased vanillin is 25–100 € kg^−1^.^[15]^

Most reviews and research articles covering the electroreduction of biobased aromatic carbonyls focus on the electrocatalytic hydrogenation and hydrogenolysis to their corresponding alcohols and alkyls, respectively.[^21^, ^22^, ^23^, ^24^] For example, 5‐HMF is electrochemically reduced to 2,5‐bis(hydroxymethyl)furan (BHMF), a precurser for the synthesis of polyester and resins^[25]^ or furfural is electrochemically reduced to furfuryl alcohol and 2‐methylfuran, which are promising candidates for biofuels.[^26^, ^27^] However, EHD of biobased aromatic carbonyls also leads to interesting compounds. The bifunctionality of the pinacol products can be exploited for the synthesis of novel renewable polymers. Moreover, the produced C_10_–C_14_ compounds from the C−C coupling by EHD offer the use as diesel drop‐in fuels for heavy duty applications, such as for aviation or in the maritime sector. Depending on the targeted application reaction conditions can be adapted accordingly to tune the selectivity towards either hydrogenation and hydrogenolysis products or C−C coupling products by EHD. This enables high flexibility and broadens up the product scope from biobased aromatic carbonyls. In this review we highlight the EHD of the biobased compounds furfural, 5‐HMF and vanillin and show potential as well as existing applications of their corresponding C−C coupling products. We only focus on their EHD in aqueous electrolytes, thus, fulfilling the requirements of a green chemistry.

## Reaction Mechanism of Electrohydrodimerization

**Scheme 1:**
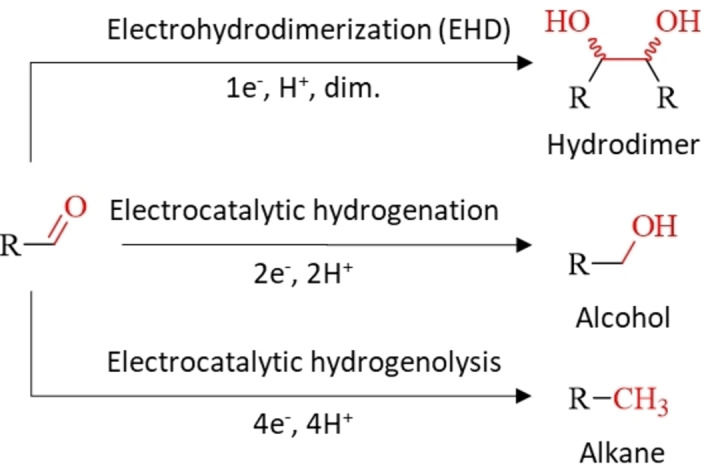
Possible reaction pathways of the electroreduction of biobased aromatic carbonyls in aqueous electrolytes.

**Scheme 2:**
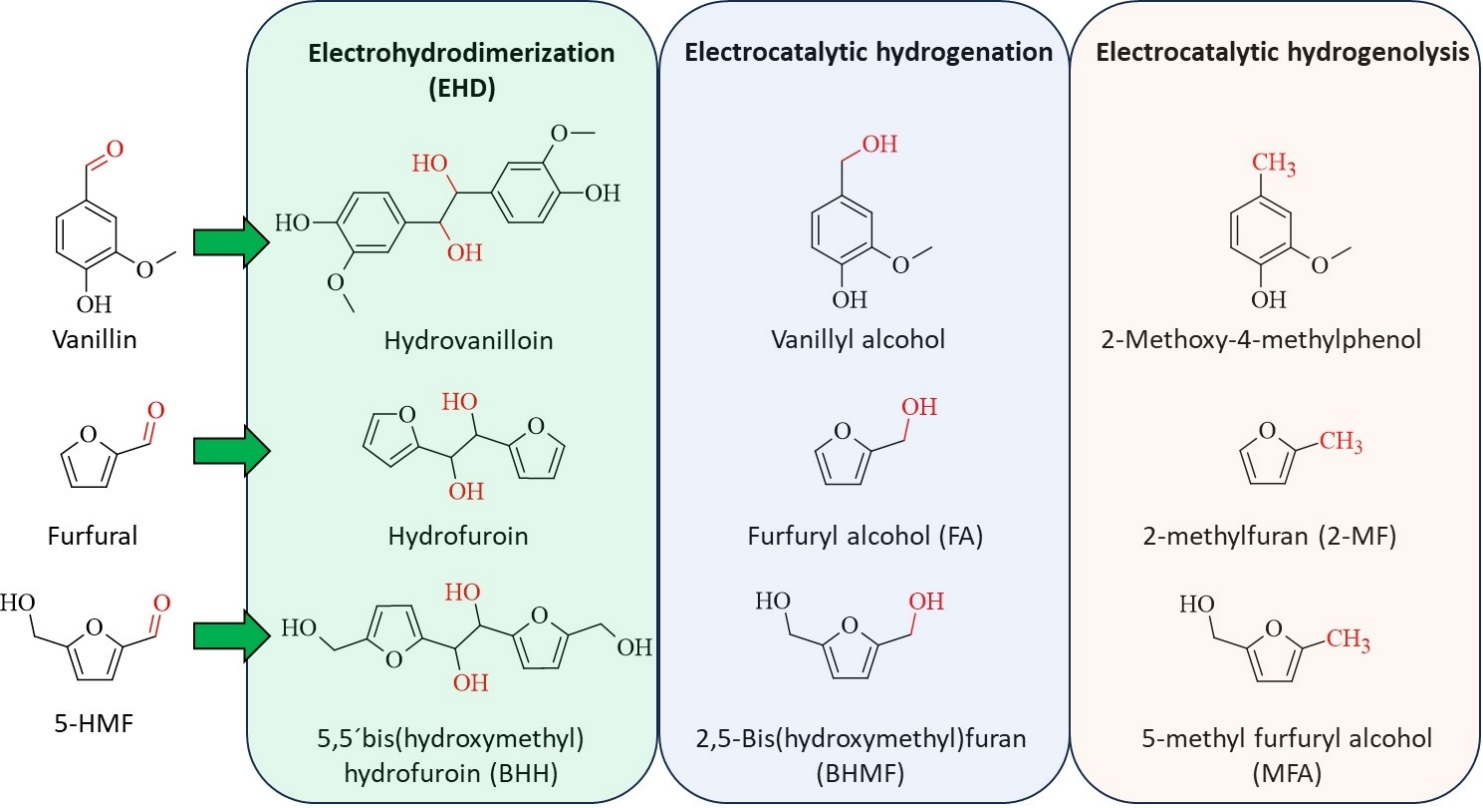
Reaction products of vanillin, furfural and 5‐HMF obtained via EHD, electrocatalytic hydrogenation and electrocatalytic hydrogenolysis.

The electroreduction of the lignocellulose‐derived aromatic carbonyls leads to different products depending on the electrochemical conditions, such as cathode material and applied potential, the chemical properties of the electrolyte as well as concentration of the substrate. In general, three main products are obtained upon reduction: (i) the hydrodimer *via* EHD, which is a 1e^−^ reduction step of the substrate followed by self‐coupling, (ii) the alcohol *via* electrocatalytic hydrogenation, which is a 2e^−^ reduction step and (iii) the alkane *via* electrocatalytic hydrogenolysis, which is a 4e^−^ reduction step (Scheme 1). The representative reaction products of vanillin, furfural and 5‐HMF obtained from these three reaction pathways are shown in Scheme 2. Moreover, the competing hydrogen evolution reaction (HER) at the cathode needs to be considered for the reaction outcome of the electroreduction of carbonyls. For acidic and alkaline media, the generation of adsorbed hydrogen (H*) via the Volmer step followed by either the Heyrovsky or the Tafel mechanism is written as follows:

Acidic media

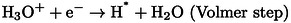




Alkaline media

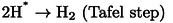




In recent years, much effort has been spent to deeply investigate the reaction mechanism of the electroreduction of biobased carbonyls. However, it should be noted that most of the studies are focused on the production of the corresponding alcohol or the alkane. Therefore, investigations were mainly carried out in acidic media favoring these targeted products over the hydrodimer.

In 2017, Chadderon et al. conducted a first thoroughly investigation of the reaction mechanisms of furfural at copper electrodes in H_2_SO_4_ based electrolyte.^[28]^ By modification of the electrode surface with organothiol self‐assembled monolayers (SAMs) they wanted to find out whether the reduction requires direct inference with the electrode surface, as the SAMs block the electrode′s surface and only outer‐sphere reaction by electron tunneling would occur. The found out that the formation of the furfural alcohol (FA) and 2‐methyl furfural (2‐MF) were inhibited, whereas no impact was observed for the production of hydrofuroin, the hydrodimer of furfural. Hence, they suggested that for hydrodimerization a ketyl radical is formed *via* an outer‐sphere mechanism followed by self‐coupling in the solution phase. Consequently, also the transfer of the required proton is carried out in the solution phase and is not originated by adsorbed hydrogen. The formation of hydrofuroin should be therefore independent of the type of electrode and insensitive to the electrode′s surface properties.^[29]^ This is also in agreement with Dixit et al. observing almost only hydrofuroin formation in alkaline media, when using either a Pt cathode at high overpotentials where H* is preferred over HER or a Ni cathode where H* is not generated effectively.^[30]^ This underlines that either blocking the surface by H* or absence of H* leads to hydrofuroin formation occurring in the solution phase. Other authors proposed that hydrofuroin and FA share a common intermediate, a ketyl radical,[^31^, ^32^] and the C−C coupling step to the hydrodimer occurs via combination of two adsorbed radicals.^[33]^ To bring light into this discussion, Liu et al. investigated the furfural reduction on Pb cathodes in acidic media tailoring the local environments, including H/D kinetic isotope effects (KIE) studies and local H_3_O^+^ and H_2_O contents, applying electron paramagnetic resonance (EPR) spectroscopy and using density functional theory (DFT) calculations.^[34]^ Their results confirm the suggested pathway *via* an outer‐sphere electron tunnel step followed by self‐coupling in the solution proposed by the earlier published study of their group by Chadderon et al.^[28]^ In contradiction to this, Mukadam et al. investigated the mechanism of furfural electroreduction on single atom Cu and Co catalysts under mild basic conditions *via* reaction order fitting and DFT calculations.^[35]^ They proposed a proton‐coupled electron transfer (PCET) as limiting reaction step leading to an adsorbed ketyl radical species on the electrode surface followed by self‐coupling in the bulk phase. Most recently, similar observations were made by Geng et al. on the furfural reduction at a combined polyoxotungstate and Cu complex catalyst using control experiments, KIE studies, electrochemical and spectral analyses, and DFT calculations.^[36]^ They proposed a synergistic catalysis effect, where furfural is reduced by the Cu center to a ketyl radical after protonation on the surface followed by self‐coupling in the solution phase.

**Scheme 3:**
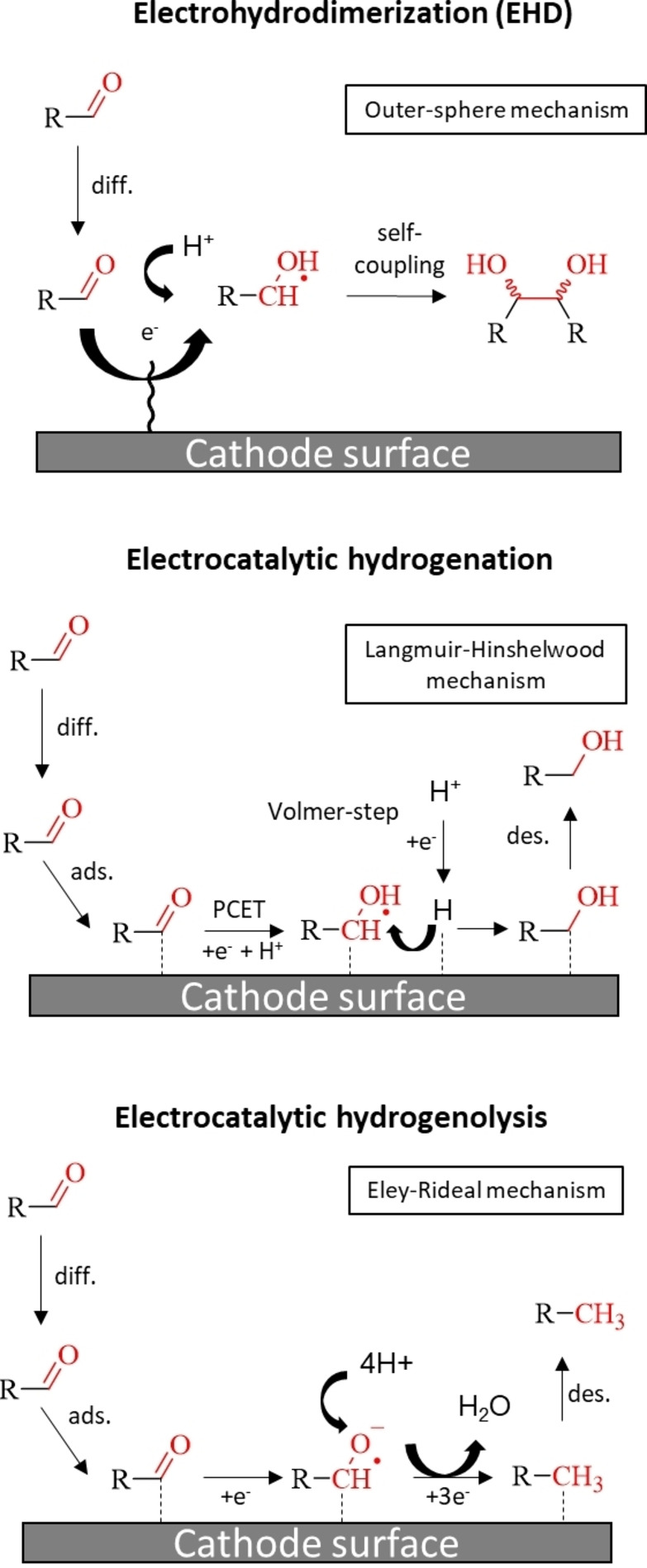
Reaction mechanism of the electrochemical reduction of carbonyls to their corresponding hydrodimeric, alcoholic and alkane products via electrohydrodimerization (EHD), electrocatalytic hydrogenation and electrocatalytic hydrogenolysis, respectively. Scheme based on investigations of furfural on Pb cathodes in acidic media.^[34]^

In contrast to the hydrofuroin production, in the study of Chadderon et al. the formation of FA and 2‐MF was heavily impacted by blocking of the electrode′s surface by SAMs, which suggests electrocatalytic hydrogenation *via* an adsorbed carbonyl substrate along the reaction mechanism.^[28]^ By conducting pathway studies comprised of cyclic voltammetry (CV) measurements as well as chronoamperometric experiments, they further revealed that FA and 2‐MF do not share a common intermediate, but rather are parallel reactions taking place on the cathode′s surface.^[28]^ To elucidate the mechanism more precisely, H/D KIE studies were conducted by Liu et al. to unravel the role of adsorbed hydrogen in comparison to hydrogen originated from the solution.^[34]^ The authors showed that formation of 2‐MF (alkane) was not affected by KIE, whereas the formation of FA (alcohol) exhibited strong dependency. Based on this result they suggested a PCET and an electron transfer (ET) as rate‐determining steps (RDS) for FA and 2‐MF production, respectively. Applying electro‐kinetic reaction order fitting they finally proposed a Langmuir‐Hinshelwood mechanism for FA, where adsorbed hydrogen generated by the Volmer‐step is involved, and an Eley‐Rideal mechanism for 2‐MF formation, where hydrogen is originated from the solution phase. Moreover, the same authors also proposed that the reaction mechanism should be transferable to other organic compounds bearing carbonyl groups, such as 5‐HMF and benzaldehyde. Scheme 3 summarizes this reaction mechanisms of carbonyl compounds to their corresponding hydrodimers, alcohols and alkanes at Pb cathodes.

For vanillin the reaction mechanism is different as the nature of the phenol group influences the stability of the radical. In 1986 Jow et al. investigated the vanillin reduction at Hg electrodes in neutral to alkaline media.^[37]^ The pH value was adjusted using sodium‐based electrolytes. In contradiction to the results of the recently published proposed mechanism for the formation of hydrofuroin on Pb cathodes, they proposed a mechanism based on electro‐kinetic reaction fitting, where the formation of the hydrodimer hydrovanilloin and vanillyl alcohol share an adsorbed metal‐ketyl (Na‐RCHO_ads_) as common intermediate. The adsorbed metal‐ketyl can either than dimerize to hydrovanilloin or a second electron transfer occurs leading to the formation of vanillyl alcohol. As hydrovanilloin was the main product at high pH values and vanillyl alcohol formation was neglectable, they suggested that the release of electrons to the benzene ring by the dissociated phenol group at high pH values leads to instability of the adsorbed metal‐ketyl favoring dimerization over the second electron transfer to vanillyl alcohol.

Moreover, the role of the cation in the electrolyte plays an important part changing not only the nature of the electrochemical double layer, but also hydration shell properties and substrate‐cation interactions. Recently, Asfia et al. investigated the role of alkali cations on the selectivity of 5‐HMF electroreduction on glassy carbon cathodes.^[38]^ The authors found that the selectivity of the electroreduction is very sensitive to the size of the cations in the supporting electrolyte and their respective concentrations. Enlarging the cation size (Li^+^ to Cs^+^) in carbonate buffers as supporting electrolytes leads to an increase of the electrocatalytic hydrogenation product BHMF, which was suggested to be due to stabilized H* species on the electrode surface by specific cation adsorption of Cs^+^ on glassy carbon. The formation of BHH *via* EHD was not affected by the size of the cation. Therefore, using smaller cation sizes suppresses electrocatalytic hydrogenation and increases the relative selectivity towards EHD.

In summary, more investigations are required to completely reveal the complex mechanism of the electroreduction of carbonyls, especially focusing on the existence of an adsorption step for the EHD on different electrode substrates, the mechanism of functionalized carbonyl compounds, such as vanillin, and the role of the electrolyte′s composition.

Nevertheless, based on the existing knowledge on the mechanism of the electroreduction of biobased aromatic carbonyls beneficial reaction conditions for either EHD or electrocatalytic hydrogenation and hydrogenolysis can be derived tuning the selectivity and Faradaic efficiencies (FE) of the respective products accordingly. The selectivity and FE depend mainly on the surface coverages of adsorbed hydrogen H* and of the adsorbed carbonyl species as well as their conversion rate.^[39]^ For the cathode material the overpotential for the HER is a crucial factor. Cathode materials exhibiting low overpotentials for the HER, such as Pt and Pt group metals, lead to high surface coverages of adsorbed hydrogen H*. Therefore, the necessary step of parallel adsorption of the carbonylic species on the surface for electrocatalytic hydrogenation and hydrogenolysis is prevented at higher current densities. Although the selectivity of EHD is not affected at these high current densities due to the outer‐sphere mechanism, as investigated by Dixit et al.,^[30]^ FEs for EHD products are very low and H_2_ is the main reaction product. When targeting electrocatalytic hydrogenation and hydrogenolysis cathode materials should exhibiting a medium overpotential for the HER to allow parallel adsorption of hydrogen and the carbonylic species. The selectivity of these electrocatalytic reactions depends also on the binding energy of the carbonyl species on the surface. For example, reduction of 5‐HMF on cathode materials, which bind weakly to 5‐HMF (In, Cd and Ag), favors the formation of the alcohol BHMF *via* electrocatalytic hydrogenation. On the other side, on cathode materials, which bind more strongly to 5‐HMF (Cu, Ni, Co and FE), the formation of the electrocatalytic hydrogenolysis product 5‐methyl furfuryl alcohol (MFA) is favored.^[40]^ As the main focus of this review is the EHD, we refer to other reviews for a deeper discussion on optimal cathode materials and reaction conditions for electrocatalytic hydrogenation and hydrogenolysis.^[24]^ When targeting EHD cathode materials with high overpotentials for the HER, such as Pb, GC, BDD, C, Zn and Hg, are the optimal choice, although the use of Hg should be avoided due to its high toxicity. The low surface coverage of adsorbed hydrogen H* suppresses electrocatalytic hydrogenation reactions as well as H_2_ formation, which leads to high selectivity and high FEs for EHD products. Table 1 summarizes the selection criteria for cathode materials depending on the targeted products. However, it should be noted that at high current densities or at respective very negative potentials still a significant amount of surface coverage of adsorbed hydrogen can be reached on high HER overpotential cathode materials leading to a significant increase in the formation of hydrogen and hydrogenation products. For example, Kloth et al. showed for the 5‐HMF electroreduction that at a very negative potential of −1 V vs. RHE at BDD and GC cathodes a significant increase of the HER and the electrocatalytic hydrogenation occurs.^[41]^ Therefore, a low polarized cathode or lower geometric current densities are also beneficial for reaching high selectivity and high FEs for EHD products. Moreover, the electrolyte composition, such as pH value and concentration of the substrate, plays a crucial role for the reaction outcome. A high pH value favors the formation of EHD products, which is due to higher kinetic barriers for the HER and hydrogenation reactions at these high pH values.^[31]^ Also, a high concentration of the carbonyl species in the electrolyte is beneficial for EHD suppressing competing reactions, such as the HER and electrocatalytic hydrogenation.^[24]^

**Table 1:** Selection criteria for cathode materials for the electroreduction of biobased aromatic carbonyls based on selectivity (main reaction pathways) and FEs towards EHD and electrocatalytic hydrogenation and hydrogenolysis.

Typical cathode materials	Overpotential for the HER	Surface coverage of adsorbed H*	Main reaction pathways for biobased aromatic carbonyls	Faradaic efficiencies
Pt, platinum group metals	Low	High	Electrohydrodimerization (EHD) at high current densities	High for HER, low for EHD
Cu, Ni, Co, Fe, In, Cd, Ag	Medium	Medium	Electrocatalytic hydrogenation and hydrogenolysis	High for electrocatalytic hydrogenation and hydrogenolysis products
Pb, GC, BDD, C, Zn, Hg	High	Low	Electrohydrodimerization (EHD)	High for EHD products

## Vanillin

### Electrochemical Reduction of Vanillin to Hydrovanilloin

**Scheme 4:**
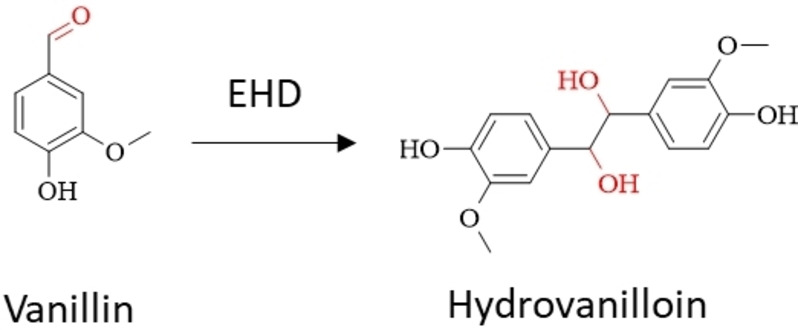
Synthesis of hydrovanilloin via EHD of vanillin.

The feasibility of the electrochemical reduction of vanillin to hydrovanilloin was first reported by I.A. Pearl in 1952 (Scheme 4).^[42]^ The electroreduction was performed in a 3‐liter beaker cell with two cylinder electrodes of a diameter of 4 cm and 14 cm for the anode and cathode, respectively, which were divided by a porous cup separator. The catholyte consisted of 1.66 M NaOH aqueous solution with a vanillin concentration of 0.66 M. A current of 3 A was maintained for 5 h, which corresponds to an applied charge of 1.17 F. The temperature of the electrolyte at the start of electrolysis was 60 °C, however, no trace heating was established, which lead to a decrease to 25 °C after the electrolysis. The exact current densities cannot be stated, as the depth of immersion of the cylinder electrodes were not mentioned. After the electrolysis hydrovanilloin was isolated with a yield of 70 % (51 g) as white solid by acidifying the electrolyte with SO_2_ followed by filtering of the precipitate. In a second experiment the yield could be increased to 78 % (86 g) by increasing the applied charge to 1.58 F (3.6 A for 8.5 h) at an adjusted catholyte comprised of 1.25 M NaOH and 0.45 M vanillin. In 1987, Jow and Chou conducted a first systematic investigation of reaction parameters on product distribution and kinetics of the cathodic reduction of vanillin.^[37]^ The cathodic reduction was conducted in an H‐type batch cell separated by a glass frit at a mercury pool cathode. The impact of pH‐value (8–14), current density (15–100 mA cm^−2^) and vanillin concentration (0.033–0.1 M) on the distribution on the two only observed reaction products hydrovanilloin and vanillyl alcohol was studied. The authors showed that with increasing pH value the production of hydrovanilloin is favored over the vanillyl alcohol generation. At pH 14 (1 M NaOH) only hydrovanilloin and at pH 8 almost only vanillyl alcohol was observed. Moreover, a high concentration of vanillin lead to high selectivity toward hydrovanilloin. Optimal reaction parameters were found to be 0.1 M vanillin in 1 M NaOH at 100 mA cm^−2^ achieving 100 % selectivity toward hydrovanilloin at a FE of 80 %. The original synthesis of hydrovanilloin by I.A. Pearl was picked up later by several authors for the production of hydrovanilloin‐based polymers using lead electrodes in alkaline media.[^43^, ^44^, ^45^, ^46^, ^47^] More recently, our group studied the influence of different cathode materials on product distributions and FEs of the electrochemical reduction of vanillin in alkaline media (1 M NaOH) in a divided H‐type batch cell.^[48]^ It was shown that Pb cathodes do not remain stable throughout electrolysis. Two cathode materials, zinc and glassy carbon (GC), also exhibiting high overpotentials for the competing HER were screened as green material alternatives to Pb and Hg by LSV and bulk electrolysis studies. Zinc was proven as stable and excellent replacement for the usually used toxic material Pb enhancing the selectivity for hydrovanilloin to almost 100 % up to high current densities of 100 mA cm^−2^ with neglectable amounts of vanillyl alcohol and hydrogen being the only noteworthy side product. FEs for hydrovanilloin for zinc cathodes varied between 55 and 85 % after an applied charge of 0.6 F depending on the current density (30–100 mA cm^−2^). Moreover, it was highlighted that the standard protocol for isolation of hydrovanilloin by acidifying the catholyte to pH 2 followed by filtration of the precipitate is sub optimal and gives space for further improvement. Maximum isolated yield of 72 % could be achieved by this method, although almost 100 % conversion to hydrovanilloin was analyzed in the crude catholyte. In a follow‐up publication of our group the transfer of the hydrovanilloin synthesis onto a plane parallel divided electrochemical flow reactor with 56 cm^2^ Zn cathodes in recirculation mode was shown.^[49]^ Promising key figures of merit with FEs up to 89 %, space‐time‐yields up to 1.13 kg l^−1^ h^−1^ and specific energy consumption down to 0.28 kWh kg^−1^ were achieved at low vanillin conversions. At high vanillin conversion of 95 % mass transport limitations of the process resulted in a decline of these figures of merit. However, using shape‐optimized turbulence promotors and model assisted process operation a space‐time‐yield of 0.48 kg l^−1^ h^−1^ at an energy consumption of 0.87 kWh kg^−1^ for 95 % vanillin conversion at a current density of 54 mA cm^−2^ could be achieved.

### Electrochemical Reduction of Divanillin to Polyvanillin

**Scheme 5:**
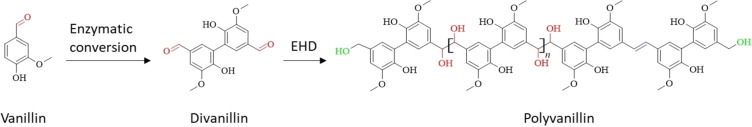
Production of polyvanillin by EHD of divanillin obtained via enzymatic conversion. The functional groups of polyvanillin generated upon EHD of divanillin are labeled in green for terminal alcohols, red for pinacols and blue for stilbens in the aliphatic region of the polymer.

In 2012, the EHD of vanillin to hydrovanilloin was picked up by Amarasekara et al. to synthesize a novel total vanillin‐based polymer called polyvanillin.^[44]^ In this first feasibility study polyvanillin was generated by EHD of divanillin (6,6′‐dihydroxy‐5,5′‐dimethoxy‐(1,1′‐biphenyl−)‐3,3′‐dicarboxyaldehyde), which is a dimeric derivative of vanillin bearing two remote carbonyl groups. Divanillin is easily accessible by enzymatic oxidation *via* horseradish peroxidase or *via* laccase in the presence of H_2_O_2_ or O_2_, respectively.[^50^, ^51^] The structure of polyvanillin was confirmed by ^1^H and ^13^C NMR and weight averaged molecular weights *M*_w_ up to 16700 g mol^−1^ were achieved. However, no calibration standard was mentioned. The polymer showed good thermal stability with a degradation onset temperature of *T*_onset,degrad_=300 °C and a 50 % weight loss temperature of *T*50 %=440 °C and is insoluble in water, alcohols and common organic solvents highlighting its possible application as high‐performance polymer. Later, our group investigated the impact of cathode materials (Zn, Pb and GC), current density and applied charge on the molecular weight distribution of polyvanillin and its molecular structure.^[48]^ Weight‐averaged molecular weights *M*_w_ up to 3400 g mol^−1^ (vs. pullulan standard) at Zn cathodes were achieved. The 2D‐NMR analytics (^13^C/^1^H, HSQC) confirmed full conversion of carbonyl groups and, besides the expected pinacol groups, terminal alcohol groups and stilbene‐like double bound systems in the aliphatic region of the polymer were observed. It was also highlighted that polyvanillin remains dissolved in the alkaline electrolyte and is not deposited on the electrode nor precipitated throughout the electrolysis due to its deprotonated phenolic groups in alkaline solution. Scheme 5 shows the reaction pathway to produce polyvanillin. Most recently, we successfully transferred the electrochemical polyvanillin production onto a divided electrochemical flow reactor in recirculation mode.^[52]^ The molecular structure of polyvanillin was confirmed to be similar to the structure obtained in the batch studies, although slightly higher molecular weights of *M*_w_=3700 g mol^−1^ and *M*_n_=2100 g mol^−1^ were achieved. Further, we showed that the reaction mechanism follows a step‐growth type polymerization. Using a dimensionless number approach, we enhanced the process reaching current densities of 54 mA cm^−2^, a space‐time‐yield of 0.206 kg l^−1^ h^−1^ and a specific energy consumption of 2.72 kWh kg^−1^ at full divanillin conversion. The process was operated at a Zn cathode at a divanillin start concentration of 0.3 M in 1 M NaOH and an isolated yield of polyvanillin of 93 % was obtained. The polymer revealed good thermal stability with a decomposition temperature of *T*_decom._=541 °C and was characterized as thermoplastic material exhibiting a glass temperature of *T*_g_=109–123 °C and a melting temperature of *T*_melting_=190–212 °C. Table 2 summarizes the most important electrochemical parameters of studies focusing on the EHD of vanillin and divanillin.

**Table 2:** Summary of electrochemical parameters of studies focusing on EHD of vanillin and divanillin to hydrovanilloin and polyvanillin, respectively.

Cathode	Electrolyte	Isolated yield/%	FE for hydrovanilloin/%	Electrochemical parameters	Reactor	Ref.
Pb	0.66 M vanillin+1.66 M NaOH/0.45 M vanillin+1.25 M NaOH	70/78	60^[a]^/49^[a]^	3 A for 5 h (1.17 F)/3.6 A for 8.5 h (1.58 F)	Divided beaker cell with cylinder electrodes (100 g scale)	[42]
Hg	0.1 M vanillin+1 M NaOH	80	80	100 mA cm^−2^ (0.6 F)	H‐type batch cell with mercury pool cathode	[37]
Pb	0.43 M vanillin+1.66 M NaOH	69	64^[a]^	−2 V vs. Ag/AgCl for 5 h (~15 mA cm^−2^, 1.08 F)	Divided beaker cell (40 g scale)	[43]
Pb	As in^[42]^	88	n.A.	n.A.	n.A.	[44]
Pb	0.165 M vanillin+1.25 M NaOH	86	n.A.	Constant cell voltage of 3, 6, 7.5, 10 and 16 V for 3 h	Divided beaker cell (1 g scale).	[45]
Zn/ Pb/ GC	0.2 M vanillin+1 M NaOH	72/72/69^[b]^	55–98^[c]^	30–100 mA cm^−2^ (0.5–2 F)	H‐type batch cell (1.5 g scale)	[48]
Zn	0.2 M vanillin+1 M NaOH	95^[d]^	15–65^[e]^	Up to 50 mA cm^−2^	Divided flow cell with recirculation (56 cm^2^)	[49]
Pb	0.175 M divanillin+1 M NaOH	91	n.A.	1.1 A using a 12 V supply for 3 h (17.6 F)	Divided beaker cell (1 g scale)	[44]
Zn/ Pb/ GC	0.1 M divanillin+1 M NaOH	58–75	n.A.	15–60 mA cm^−2^ (2–8 F)	H‐type batch cell (1.5 g scale)	[48]
Zn	0.3 M divanillin+1 M NaOH	93	25	54 mA cm^−2^ (8 F)	Divided flow cell with recirculation (56 cm^2^)	[52]

[a] Calculated on isolated yield. [b] All after 2 F at 30 mA cm^−2^. [c] All after 0.6 F. [d] Not isolated. [e] At 95 % conversion.

### Follow‐Up Reactions for Valorization of Hydrovanilloin

Besides the direct electrosynthesis of a polymer using EHD as in the case of polyvanillin, hydrovanilloin was used as building block or intermediate in many classical polymer syntheses. The multifunctional compound hydrovanilloin bearing two phenolic and two aliphatic OH groups offers broad possibilities for the production of biobased polymers. In 2015, Harvey et al. have prepared a cyanate ester resins and polycarbonate thermoplastics from a hydrovanilloin derivate.^[43]^ The intermediate bisphenol precurser was produced either *via* hydrovanilloin generated electrochemically or *via* the McMurry coupling reaction. In case of the electrochemical produced hydrovanilloin, the vicinal OH‐groups in the aliphatic region were reduced to either the corresponding stilbene‐like double bound systems or to their alkanes and further converted to their cyanate esters. Finally, thermoset polymer materials were produced by thermal cyclotrimerization to form polycyanurates. Moreover, the reduced bisphenolic compound was used in the synthesis of a polycarbonate by transesterification with diphenyl carbonate. The produced polymers from the reduced bisphenolic compound showed moderate to excellent thermal behavior with glass temperatures of *T*_g_=202 °C and *T*_g_=86 °C for the cyanate ester resin and the polycarbonate, respectively. In 2016, Amarasekara et al. prepared a hydrovanilloin‐formaldehyde with moderate molecular weights of *M*_n_≈10000 g mol^−1^ and high yields up to 91 %.^[45]^ The polymer showed good thermal stability up to 360 °C in air. Later, the same working group synthesized a hydrovanilloin‐based epoxy resins highlighting its possibility as sustainable substitution to bisphenol A.^[46]^ In a first approach, a hydrovanilloin‐diglycidyl ether phenoxy resin was generated by conversion of the disodium salt of hydrovanilloin with equivalent amounts of epichlorohydrin. Excellent yields of 87 % and a *T*_g_ of 135 °C were achieved. In a second step, a curable oligomer with an average number of repeating units of 2.1 was produced using an excess of epichlorohydrin. The oligomer was then cured with aliphatic diamines resulting in hard epoxy resins with *T*_g_ ranging between 116 and 149 °C depending on the used amine curing agent. More recently, as continuation of their work the same group synthesized a hydrovanilloin‐based urethane.^[47]^ As a first step, hydrovanilloin was converted with equivalent amounts of diisocyanates using a tertiary amine catalyst. The resulting cross‐linked *poly*(hydrovanilloin‐urethane) showed molecular weights of *M*_n_≈25000 g mol^−1^ and *T*_g_ values between 121–172 °C depending on the diisocyanate compound. In a second approach, a softer polymer was generated by first converting the diisocyanates with polyethylene‐glycol 400 followed by polymerizing these pre‐polymers with hydrovanilloin. The *poly*(hydrovanilloin‐ethylene glycol‐urethane) showed better thermal stability, however no distinct glass transition was observed.

**Scheme 6:**
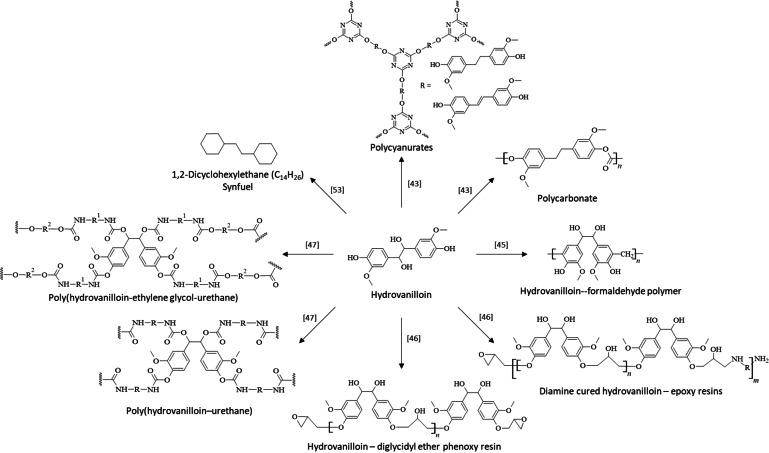
Valorization routes of hydrovanilloin to different polymers and a long‐chained synfuel for aviation and heavy‐duty applications.

Another valorization strategy is using hydrovanilloin as precurser for the production of drop‐in synfuels in heavy duty applications, where electrification is not technically possible or economically reasonable. The carbon‐chain elongation of vanillin to hydrovanilloin enables the generation of diesel range fuels, however, ring saturation and deep deoxygenation is required to reach acceptable cetane numbers.^[27]^ In 2012, Huang et al. showed in a first feasibility study the production of a C_14_H_26_ alkane by hydrodeoxygenation of hydrovanilloin with a low oxygen ratio of 2–2.5 % fulfilling the diesel fuels requirements.^[53]^ However, more characterization is required targeting the combustion behavior and fuel properties (freezing point, viscosity, char formation…) to check, for example, if the synfuel fulfills the strict requirements for ‘drop‐in’ Jet A fuels for aviation applications. The valorization strategies of hydrovanilloin to polymer and fuels are summarized in Scheme 6.

## Furfural

### Electrochemical Reduction of Furfural to Hydrofuroin

**Scheme 7:**
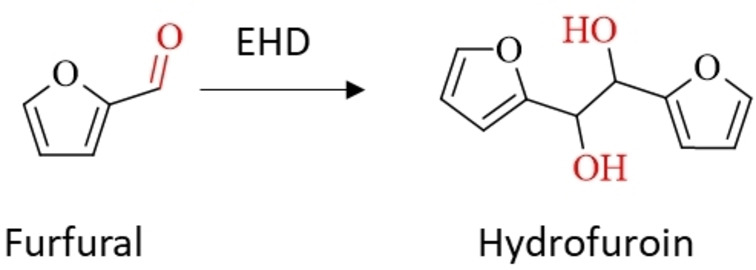
Synthesis of hydrofuroin via EHD of furfural.

The electroreduction of furfural to hydrofuroin was first described in 1939 by W.C. Albert and A. Lowy in a divided batch cell (Scheme 7).^[54]^ The effect of various parameters, such as cathode material (Pb, Zn, Zn amalgam, Cd, Hg, Ni, Cu and Pt), temperature, current density and pH value was investigated. When using strong acids or bases as supporting electrolyte undesired resinifying of furfural was observed at moderate concentrations due to its chemical instability. Using buffered solutions to maintain nearly neutral media was found to mitigate resinifying of furfural during electrolysis leading to the formation of electroreduction products. A maximum isolated yield of 63 % of hydrofuroin was achieved under optimized parameters of using a Pb cathode, a 0.74 M KH_2_PO_4_ aqueous solution as supporting electrolyte, a furfural concentration of 0.9 M and applying a charge of 1 F at a current density of 5 mA cm^−2^. The described side products were resin and furfuryl alcohol. Later in 2013, P. Nilges and U. Schröder investigated the electrocatalytic hydrogeneration of furfural targeting the 4 e^−^ reduction product 2‐MF in an H‐type batch cell.^[55]^ They investigated different cathode materials (Cu, Ni, Pt, C, Fe, Pb and Al) in 0.5 M H_2_SO_4_ water‐acetonitrile mixture as supporting electrolyte. Among the tested materials Cu showed the highest selectivity for 2‐MF. As a side result of their study, they also showed that high selectivity for hydrofuroin can be achieved at C, Fe, Pb and Al cathodes. Especially, Al showed almost quantitative yields for hydrofuroin with no FA or 2‐MF formation observed. Resinous products were not described. However, the authors did not further exploit this result in their study. Within the mechanistic study of Chadderdon et al. potentiostatic electrolysis at Pb cathodes were used to underline their suggestion that cathode materials exhibiting a high overpotential for the competing HER suppress the formation of electrocatalytic hydrogenation products (FA and 2‐MF).^[28]^ As expected, neglectable amounts of electrocatalytic hydrogenation products were observed in a potentiostatic electrolysis experiment after 1 h at −0.55 V vs. RHE at pH 0.5 and pH 3 electrolytes. FEs of 34 % and 38 % for hydrofuroin were found at pH 0.5 and pH 3, respectively. Besides hydrofuroin, resinous products and unidentified peaks in the chromatographic analysis were observed. Dixit et al. investigated the electrochemical reduction of furfural in a divided H‐type batch cell in alkaline media (0.5 M NaOH) at moderate concentrated solutions of furfural of 10–100 mM.^[30]^ Different cathode catalysts (Ni‐foam, Cu, Pt, NPNi/Ni‐foam and Cu‐NPNi/Ni‐foam) were investigated with respect to their selectivity and reaction rate toward FA and hydrofuroin. The highest amounts of FA and hydrofuroin were found on Cu‐NPNi/Ni‐foam. Reaction rates of 135 μmol h^−1^ cm^−2^ at a selectivity of 14 % for FA and 341 μmol h^−1^ cm^−2^ at a selectivity of 69 % for hydrofuroin was reached after 1 h electrolysis at −0.45 V vs. RHE with a furfural concentration of 100 mM. The total conversion of furfural was 66 %. Decreasing numbers were obtained at lower furfural concentrations, however, the conversion of furfural increased to 75 % at 10 mM. In a more general study about coupling of aromatic carbonyls to their corresponding hydrodimers Liu et al. investigated the generation of hydrofuroin within the substrate scope.^[56]^ A yield of 97 % was reached at a carbon paper cathode in 1 M KOH in an H‐type batch cell. The authors managed to transfer the production of the hydrodimer onto a flow reactor without an anion or cation exchange membrane, but using a porous carbon membrane, which is simultaneously the cathode. Although the setup was successfully tested for the coupling of benzaldehyde, the electroreduction of furfural was not investigated in this setup. Shang et al. transferred the EHD of furfural to hydrofuroin onto a divided electrochemical flow reactor in filter press configuration.^[31]^ 10 stacked carbon paper sheets (each 1 cm×5 cm) served as inexpensive cathode material. A low furfural concentration of 10 mM in an alkaline electrolyte of 0.1 M KOH was used. Anode and cathode side were separated by an anion exchange membrane. As counter reaction on the anode side the oxygen evolution reaction at a Ni‐based foam electrode was chosen. Optimal parameters for hydrofuroin regarding energy efficiency and productivity were obtained by varying the cell voltage in constant cell voltage operation mode. A hydrofuroin yield of 89 % with an excellent FE of 82 % in single pass of the flow electrolyzer was achieved at a cell voltage of −2.1 V. At this cell voltage geometric current densities of ≈2 mA cm^−2^ with a production rate of 0.13 mmol h^−1^ were derived. Later, Cao et al. developed a paired electrosynthesis in a divided electrochemical microflow reactor.^[57]^ On the anode side the oxidation of furfural to 2(5*H*)‐furanone and on the cathode side the reduction of furfural to FA and hydrofuroin was employed. As supporting electrolyte and mediator for the anodic half reaction NaBr in H_2_O was used. The authors emphasized the use of a membrane to prevent any competing reactions at the corresponding counter electrodes, which would result in low yields and selectivity. Varying the cathode material and the applied cell voltage in constant cell voltage operation mode, a maximum yield of 71 % for hydrofuroin was achieved at a cell voltage of −2.9 V at a Pb cathode and a furfural concentration of 0.1 M. Additionally, a yield of 20 % of furfuryl alcohol on the cathode side and a yield of 77 % of 2(5*H*)‐furanone at a graphite anode was obtained in the same experiment. Increasing the furfural concentration up to 0.6 M lead only to a minor decrease of the hydrofuroin yield of ≈60 %, however, improving the productivity significantly. In 2022, Huang et al. demonstrated how the structural phase distribution of a MoS_2_ transition metal dichalcogenides (TMDs) catalyst impacts the reaction outcome of the electroreduction of furfural.^[58]^ Two different phases, 1T and 2H, were prepared and tested for their selectivity for electrocatalytic hydrogenation and EHD. A constant potential electrolysis was conducted at −0.272 V vs. RHE for 1.6 h and a methanol‐water mixture with 0.4 M Na_2_B_4_O_7_ as supporting electrolyte and a furfural concentration of 20 mM was used. On the 2H MoS_2_ cathode a selectivity of 43 % was achieved for hydrofuroin at a furfural conversion of 48 %, whereas neglectable amounts of hydrofuroin were found on the 1T phase MoS_2_. The study showed that on the 1T phase the ability to generate adsorbed hydrogen was facilitated in comparison to the 2H phase catalyst, which leads to an increase of electrocatalytic hydrogenation and lowered the selectivity towards EHD. Although the hydrofuroin selectivity and the furfural conversion were not reasonable high, this study shows that tuning of the catalyst structure can affect the reaction outcome by changing the amount of adsorbed hydrogen on the surface, which agrees with the proposed mechanism. Xu et al. investigated the electrochemical hydrogenation of furfural to furfuryl alcohol on Cu catalysts supported on N‐doped hierarchically porous carbon (15 %‐Cu/NC_900_).^[59]^ Although the authors focused on the production of furfuryl alcohol, they found a high sensitivity of the applied potential on the selectivity of the reaction. Using an H‐type batch cell a high hydrofuroin selectivity of 56 % at 93 % furfural conversion was obtained upon electroreduction of a 30 mM furfural +1 M KOH electrolyte at a potential of −0.45 V vs. RHE for 4 h. Interestingly, at more positive potentials (−0.35 V vs. RHE) no hydrofuroin formation was observed. The authors discussed that at too negative potentials a mismatch of adsorbed hydrogen and furfuryl substrate on the Cu electrode occurs decreasing electrocatalytic hydrogenation selectivity and favoring EHD, which aligns with the proposed mechanism. Recently, Temnikova et al. studied the furfural reduction to hydrofuroin in organic media to circumvent solubility issues and to suppress the competing HER in aqueous electrolytes.^[60]^ Different solvents, cathode materials and conductive salts were screened with regard to activity, selectivity, conversion and FE toward hydrofuroin generation. Acetonitrile with a small amount of water (5 %) promoting hydrofuroin production was shown as best choice among the tested solvents (MeCN, MeNO_2_, DMSO, DMF). Polycrystalline Pd and Cu cathodes showed the best performance, whereby Pd achieved a higher selectivity at higher overpotentials and currents. Oxidation of the tested tetraalkylammonium conductive salts was the counter reaction on the anode, which interfered with the hydrofuroin generation at the cathode, as an undivided cell was used. However, tetrabutyl‐ammonium‐iodide was shown as excellent choice achieving high yields and FEs for hydrofuroin even using an undivided cell configuration. Full conversion of furfural (50 mM) was reached with hydrofuroin yields of 94.5 % and a FE of 72 %. Another approach using Cu and Co single atom electrocatalysts, which were supported on carbon electrodes by non‐covalent adsorption, for the EHD of furfural was reported by Mukadam et al.^[35]^ In detail, Co and Cu phthalocyanines (Pc) adsorbed onto multi‐walled carbon nanotubes (MWCNTs) were used as electrocatalysts. The electrosynthesis was carried out in a divided batch cell at a mild pH of 10 (0.1 M KHCO_3_) at a furfural concentration of 20 mM. A high FE for hydrofuroin of 73 % and 77 % was obtained upon potentiostatic electrolysis of −0.5 V vs. RHE for 1 h for CoPc and CuPC, respectively. Rabet et al. investigated the effect of acetic acid in the electrolyte on the EHD of furfural to hydrofuroin to simulate impurities of crude furfural.^[61]^ An acidic electrolyte comprised of 0.1 M H_2_SO_4_ and 25 % CH_3_CN was used with addition of either 50 mM furfural or 42 mM furfural and 8 mM acetic acid. The electrochemical studies were conducted in an H‐type batch cell with Pb and laser‐structured Pb cathodes. Although only a highest selectivity of ≈30 % for hydrofuroin with a production rate of 75 mmol h^−1^ cm^−2^ was obtained at a constant potential of −0.985 V vs. Ag/AgCl at the laser‐structured Pb cathode without addition of acetic acid, structuring of the Pb cathode enhanced the selectivity and productivity slightly and addition of acetic acid did not affect the hydrofuroin formation significantly. The low production rate and selectivity was discussed to be originated from the dominance of the HER in the acidic electrolyte. However, this study highlights the use of a crude furfural feedstock, which would be relevant for application of this process on an industrial scale. More recently, EHD of furfural to hydrofuroin was realized by Geng et al. at neutral pH at a Cu‐PW_12_/CC cathode.^[36]^ The cathode was fabricated by combining a Cu‐pyrazine complex with Keggin‐type PW_12_O_40_, and an electron sponge. At a furfural concentration of 50 mM in an 0.4 M NaH_2_PO_4_/Na_2_HPO_4_ methanol‐water (1 : 4, v/v) electrolyte a selectivity of 92 % and a FE of 85.1 % at 99 % conversion was reached at constant potential electrolysis of −0.76 V vs. RHE. The authors explained this remarkable result by synergistic catalysis effects between the Cu center and PW_12_. Table 3 summarizes the most important electrochemical parameters of studies focusing on the EHD of furfural to hydrofuroin.

**Table 3:** Summary of electrochemical parameters of studies focusing on EHD of furfural to hydrofuroin.

Cathode	Electrolyte	Yield/%	FE for hydrofuroin/%	Electrochemical parameters	Reactor	Ref.
Pb	0.9 M furfural+0.74 M KH_2_PO_4_	63^[a]^	62	5 mA cm^−2^ (1 F)	Divided beaker cell with cylinder electrodes (86 g scale)	[54]
Al	Furfural concentration unknown+0.5 M H_2_SO_4_ (water+acetonitrile)	n.A.	n.A.	10 mA cm^−2^	H‐type batch cell – 100 % selectivity for hydrofuroin	[55]
Pb	0.05 M furfural+0.5 M H_2_SO_4_ (pH=0.5) or 0.5 M sulfate solutions (pH=3) with 25 % v/v CH_3_CN as cosolvent	n.A.	34–38	−0.55 V vs. RHE for 1 h (<1 mA cm^−2^)	H‐type batch cell	[28]
Cu‐NPNI/Ni‐foam	0.1 M furfural+0.5 M NaOH	45	n.A.	−0.45 V vs. RHE for 1 h	H‐type batch cell	[30]
C	0.016 M furfural+1 M KOH	97	n.A.	Constant potential^[b]^	H‐type batch cell	[56]
C	10 mM furfural+0.1 M KOH	89	82	−2.1 V cell voltage (≈2 mA cm^−2^)	Divided filter press flow reactor with 5 cm^2^ geometric electrode area	[31]
Pb	0.1–0.6 M furfural+0.1 M NaBr	60–71	n.A.	−2.9 V cell voltage	Divided microflow reactor	[57]
2H‐rich MoS_2_	0.02 M furfural+0.4 M Na_2_B_4_O_7_ in water with 25 % v/v MeOH	20	15	−0.272 V vs. RHE for 1.6 h	Divided batch cell	[58]
15 %‐Cu/NC_900_	0.03 M furfural+1 M KOH	52	n.A.	−0.45 V vs. RHE for 4 h	H‐type batch cell	[59]
Pd	0.05 M furfural+0.1 M TBAI in MeCN with 5 % of water	95	72	−1.7 V vs. Ag/Ag^+^ until full conversion	Undivided batch cell	[60]
CoPc/CuPC	0.05 M furfural+0.1 M KHCO_3_	n.A.	73/77	−0.5 V vs. RHE for 1 h	Divided batch cell	[35]
Laser‐structured Pb	0.05 M furfural or 0.042 M furfural+0.008 M acetic acid in 0.1 M H_2_SO_4_ with 25 % v/v CH_3_CN as cosolvent	n.A.^[c]^	n.A.^[c]^	−0.985 V vs. Ag/AgCl for 1 h	H‐type batch cell	[61]
Cu‐PW_12_/CC	0.05 M furfural in 0.4 M NaH_2_PO_4_/Na_2_HPO_4_ CH_3_OH/H_2_O=1 : 4 (v/v)	91	85	−0.76 V vs. RHE for 6 h	H‐type batch cell and filter press flow reactor	[36]

[a] Isolated yield. [b] No exact potential stated. [c] Only production rate and selectivity stated.

### Follow‐Up Reactions for Valorization of Hydrofuroin

**Scheme 8:**
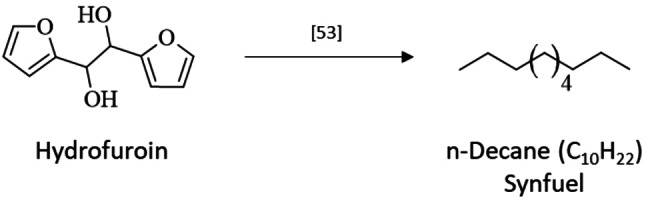
Valorization route of hydrofuroin to a long‐chained synfuel for aviation and heavy‐duty applications.

Hydrofuroin is considered as biofuel precurser for aviation and heavy‐duty applications.[^31^, ^53^, ^62^] The C−C coupling of the C_5_ compound furfural to the C_10_ compound hydrofuroin enables it for use as diesel type fuel. However, similar to hydrovanilloin a hydrodesoxygenation step is required to get rid of the unsaturated aromatic ring, to lower the oxygen content and to enhance the heating value of the fuel. Huang et al. showed the production of C_10_H_22_ from hydrofuroin with an alkane fraction up to 84 % and an oxygen ratio down to 9 % (Scheme 8).^[53]^ The low price of furfural and its already high global production rate renders hydrofuroin as a promising candidate as jet fuel precurser. However, a thorough characterization, improvements of the processes regarding scale and techno‐economic evaluations are still missing to elucidate its practical feasibility.

To the best of our knowledge, follow‐up reactions of hydrofuroin to polymers have not been investigated, as no further exploitable functional groups are attached to the furanic rings of hydrofuroin.

## 5‐HMF

### Electrochemical Reduction of 5‐HMF to BHH

**Scheme 9:**
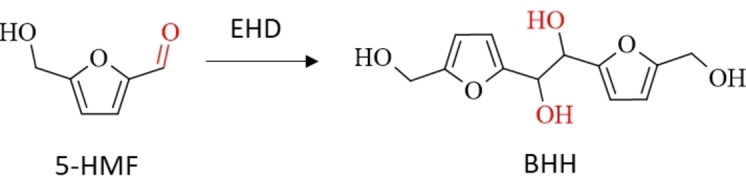
Synthesis of BHH via EHD of 5‐HMF.

In comparison to furfural, 5‐HMF is a very recently derived biobased platform chemical. Due to its multifunctional structure 5‐HMF rapidly gained much interest for the preparation of biobased value‐added compounds and fuels.^[63]^ The electroreduction of 5‐HMF was first described by Kwon et al. in 2013, however, focusing on the hydrogenation to BHMF and other hydrogenated products, such as 2,5‐dimethylfuran (DMF).^[64]^ Later, also targeting hydrogenation of 5‐HMF Roylance et al. mentioned C−C coupling side products, such as dimers or polymers, on the basis of NMR data, although BHH was not stated explicitly. The formation of BHH via EHD of 5‐HMF (Scheme 9) was first described by Chadderdon et al. in 2019 as side product on the cathode side in a paired electrosynthesis combining anodic oxidation of 5‐HMF to 2,5‐furandicarboxylic acid (FDCA) and cathodic reduction of 5‐HMF to BHMF.^[65]^ The synthesis was conducted in a H‐type batch cell in pH 9.2 borate buffer. Ag‐based electrocatalysts, polycrystalline and supported on carbon, were used as cathode materials. The authors showed that the product distribution was very sensitive to the applied potential and a relevant selectivity of ≈20 % for BHH formation was only observed at very low overpotentials of −1.15 V vs. Ag/AgCl with BHMF being the main product. However, at these low overpotentials 5‐HMF conversion was very low (<5 %) after 30 min of reaction time and 20 mM starting concentration. Increasing the starting concentration of 5‐HMF to 50 mM lead to an increase of the BHH selectivity to ≈40 % for Ag (polycrystalline), however, 5‐HMF conversions still remained low at 10 % at −1.2 V vs. Ag/AgCl. Interestingly, bulk electrolysis was performed only on the carbon black modified carbon paper support (CB‐CP) to decouple the contribution of the cathode materials to the reaction outcome. The selectivity/FE for BHH remained stable at ≈30 % between −1.2 and −1.5 V vs. Ag/AgCl, but significant 5‐HMF conversions of 50 % were reached at −1.5 V vs. RHE. Only neglectable amounts of BHMF were observed with other unidentified dimers or oligomers being the remaining reaction products. Based on CV studies the authors suggested that these other unidentified dimers and oligomers form upon radical combination processes induced by a one‐electron transfer to 5‐HMF, which not reacted to BHH. Later, several authors confirmed the formation BHH and oligomers as side product on Ag‐based cathodes in pH 9.2 borate buffer in BHMF production.[^66^, ^67^, ^68^, ^69^] The first study focusing on the electrochemical reduction of 5‐HMF to BHH was conducted by Kloth et al. in 2021.^[41]^ Different carbon materials (Graphite, glassy carbon (GC) and boron‐doped‐diamond (BDD)) were investigated in pH 9.2 carbonate buffer in a divided flow cell setup in recirculation mode. Applied potential, initial HMF concentration and type of carbon for cathode were optimized with regard to BHH yield. On BDDs the highest selectivity was obtained for BHH with low impact of the applied potential, which renders them as optimal choice when no precise process control is feasible. However, low activity for HMF conversion and its high price were considered as major drawbacks for this carbon material. On GC a higher activity was observed, but high selectivity of 35 % for BHH was only observed at low overpotentials down to −0.8 V vs. RHE. At potentials below −0.8 V vs. RHE significant amounts of H_2_ and BHMF were found decreasing the selectivity for BHH. An increase of the concentration of 5‐HMF was shown to suppress BHMF formation. The highest FE of 42 % for BHH was achieved at the highest tested starting concentration of 100 mM 5‐HMF after 2 h electrolysis time at −0.8 V vs. RHE (45 % 5‐HMF conversion). Almost similar electrochemical behavior to GC was found for graphite with slightly higher amounts of the by‐products H_2_ and BHMF, however, experiments were only performed at 10 mM starting concentration of 5‐HMF. However, it should be noted that the major part of the reaction products was stated as unknown with a combined selectivity of up to 71 %. Similar to earlier observations by other authors electrochemically induced reactions to oligomers (humins) and other dimerization products were proposed as reaction pathways toward these reaction products. Recently, Wu et al. achieved the highest selectivity for BHH of 70 % at TiO_2_ cathodes at a constant potential electrolysis at −0.6 V vs. RHE (1F) in a pH 7 phosphate buffer.^[70]^ A FE of ≈50 % was reached with this method. No significant decrease was observed in 8 consecutive electrolysis cycles showing the cathode′s stability. Decreasing the applied potential only slightly decreased the selectivity reaching 50 % for BHH at −0.75 V vs. RHE. Additionally, the authors stated to achieve a selectivity of 95 % for BHH at pH 13, however, the exact buffer system was not stated.

Table 4 summarizes the most important electrochemical parameters of studies focusing on the EHD of 5‐HMF to BHH.

**Table 4:** Summary of electrochemical parameters of studies focusing on EHD of 5‐HMF to BHH.

Cathode	Electrolyte	Yield/%	FE for BHH/%	Electrochemical parameters	Reactor	Ref.
CB‐CP	20 mM 5‐HMF in pH 9.2 borate buffer	17	35	−1.5 V vs. Ag/AgCl for 0.5 h	Divided H‐type batch cell	[65]
BDD GraphiteGC GC	10 mM 5‐HMF in pH 9.2 carbonate buffer100 mM 5‐HMF	17 14 17 13	33 24 36 42	−0.8 V vs. RHE for 2 h	Divided flow cell in recirculation mode (4 cm^2^ electrode area)	[41]
TiO_2_	20 mM 5‐HMF in pH 7 phosphate buffer 20 mM 5‐HMF in pH 13 buffer	n.A. n.A.	≈50 n.A.	−0.6 V vs. RHE (19.3 C/1 F)	Batch cell^[a]^ Selectivity of 70 % for BHH at pH 7 Selectivity of 95& for BHH at pH 13	[70]

[a] Not stated if divided or undivided.

### Follow‐up Reactions for Valorization of BHH

**Scheme 10:**
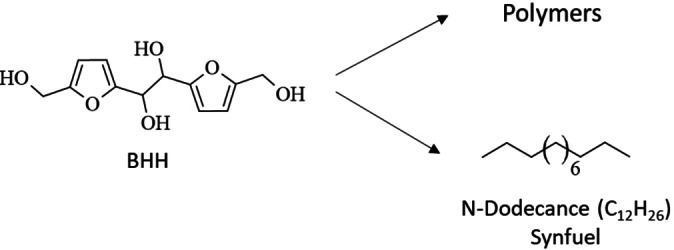
Proposed valorization routes of BHH to a long‐chained synfuel for aviation and heavy‐duty applications and to renewable polymers.

Due to the bis‐/multi‐ functional structure of BHH the compound should be useable in the synthesis of biobased polymers by exploiting its −OH functionalities. However, to the best of our knowledge no studies have been published yet using BHH for a follow‐up biobased polymer synthesis. Moreover, the carbon chain‐elongation from C_6_ (5‐HMF) to C_12_ (BHH) renders the product as biofuel precurser for aviation and heavy‐duty applications.^[41]^ Until now, BHH has not been further converted by a hydrodesoxygenation step, which is a required upgrading step for using the compound as drop‐in fuel. However, Huang et al. hydrodesoxygenized a close derivative of BHH, namely the hydrodimer of 5‐methylfurfural, to synthesize a C_12_H_26_ fuel with an alkane fraction up to 86 % at an oxygen content of under 5 %. Proposed application routes for BHH are shown in Scheme 10.

## Discussion

Although mainly electrocatalytic hydrogenation and hydrogenolysis of biobased aromatic carbonyls has been investigated over the past years, much progress has also been made recently for the EHD of vanillin, furfural and 5‐HMF to hydrovanilloin, hydrofuroin and BHH, respectively, exploring new synthetic routes for their valorization. Besides their interesting properties such as their bifunctionality for subsequent polymer synthesis or high carbon number as precurser for long‐chained synfuel synthesis, the electroreduction itself offers unique advantages, such as choice of cathode material and electrolyte composition.

In detail, low‐cost carbon‐based cathode materials, such as graphite or carbon paper, offer high selectivity towards EHD products and suppress competing reactions such as the HER leading to high FEs for EHD reactions. Moreover, they are commercially available as 3‐dimensional electrodes, for example as carbon felts, and are already used at large scale e. g. in redox‐flow batteries.^[71]^ Also, Pb was chosen in many investigations as cathode material due to its very high overpotential for the competing HER. However, although Pb is an often‐used cathode material, its stability throughout the electrolysis needs to be proven, as cathodic corrosion of Pb cathodes is a known phenomenon.[^48^, ^72^] For example, lead bronzes can be considered as alternative for Pb and tested for the EHD.^[72]^ Zn as non‐toxic and cheap metal should also be noted as interesting cathode material for EHD showing very high selectivity and FEs for the hydrovanilloin synthesis.^[48]^ In contrast to that, typical cathode materials for the electrocatalytic hydrogenation and hydrogenolysis are either expensive, such as Ni, Cu and Ag or are categorized as critical raw materials by the EU, such as Co or In.^[73]^

Further, a high alkaline pH value of the electrolyte favors EHD, whereas for electrocatalytic hydrogenation and hydrogenolysis usually acid electrolytes are selected. This facilitates material choice for an electrochemical flow reactor setup due to less corrosive properties of alkaline electrolytes and, especially, enables the use of non‐noble metals for the counter reaction at the anode (assuming also alkaline media at the anode), such as Ni‐based anodes for the oxygen evolution reaction (OER) in alkaline media.^[74]^

As discussed in the mechanistic analysis, a high concentration of the aromatic carbonyl also favors selectivity towards EHD over electrocatalytic hydrogenation and hydrogenolysis.^[24]^ This leads to higher current densities and, therefore, a high production rate. However, stability issues of the compounds need to be considered leading to chemical side reactions such as formation of humins or base‐catalyzed Cannizzaro reactions.[^75^, ^76^] These aromatic compounds bearing an aldehyde group are unstable under the highly alkaline conditions and they are decomposing even faster at high concentration.^[76]^

On the contrary side, high selectivity and FEs towards EHD products are only achieved at less‐polarized cathodes or at respective lower current densities thus limiting the productivity. Even on high HER overpotential cathodes, at very negative potentials competing reactions originated from adsorbed hydrogen (HER and electrocatalytic hydrogenation) occur, which was shown in the mentioned studies.[^41^, ^48^]

Therefore, a transfer to an electrochemical flow reactor is a crucial step for scaling up the productivity of EHD. For example, the space‐time‐yield *STY* of an electrochemical flow reactor under stationary operation is calculated by

(1) $$STY\;\left[kg\;l^{-1}h^{-1}\right]=\;\frac{M^{*}FE^{*}j^{*}A_{v}}{z^{*}F}\;$$

where, *M* is the molar mass of the product, *FE* the Faradaic efficiency, *j* the current density, *A*_v_ the ratio of electrode area to electrolyte volume, *z* the number of transferred electrons and F the Faraday constant (96485 As mol^−1^).^[77]^ In batch cells the *A*_v_ value typically not exceeds a value of 0.25 cm^−1^,^[78]^ whereas *A*_v_ values of >50 cm^−1^ are easily achieved in flow cells with porous electrodes increasing the productivity significantly.^[79]^ Moreover, using these porous 3‐dimensional electrodes reasonable absolute currents at low geometric current densities *j* can still be reached leading to a low polarization of the cathode. In such a way, the selectivity towards EHD products can be increased. Further, the specific energy consumption *E*_s_ is an important value for calculation the production costs of an electrochemical process, which is calculated under stationary operation by

(2) $$E_{S}\;\left[kWh\;kg^{-1}\right]=\;\frac{U_{cell}^{*}z^{*}F}{FE^{*}M}$$

where *U*_cell_ is the cell voltage.^[77]^ The cell voltage depends not only on the overpotentials of anode and cathode, but also on the Ohmic resistance of the cell. A reactor design with a small anode to cathode distance is desired, which is realized in compact flow cell setups, such as a filter‐press type flow reactor.^[80]^ Further, a low number of transferred electrons (*z*=2 for EHD products) and a high molar mass of the EHD products (*M*_Hydrovanilloin_=306 g mol^−1^, *M*_Hydrofuroin_=194 g mol^−1^, *M*_BHH_=254 g mol^−1^) are beneficial for a high space‐time‐yield and a low specific energy consumption. For example, assuming a reasonable *FE* of 70 % and a cell voltage of 2.5 V consisting of a cathode potential of −0.6 V vs. RHE, an anode potential of 1.5 V vs. RHE for the OER and an estimated Ohmic drop of 0.4 V, specific energy consumptions of 0.63, 0.99 and 0.75 kWh kg^−1^ are obtained for hydrovanilloin, hydrofuroin and BHH, respectively. With an assumed electricity price of 0.02 € kWh^−1^ production costs as low as 0.013, 0.020 and 0.015 € kg^−1^ are calculated for hydrovanilloin, hydrofuroin and BHH, respectively, neglecting other operational costs, such as pumps or workup of the electrolyte.^[7]^ Moreover, the stability problem of the aromatic biobased carbonyls can be solved by operating a single pass flow reactor setup, in which the pH value is adjusted close before the inlet of the flow cell limiting the residence time of the instable compounds under alkaline conditions. As a result, high concentrations of substrates (vanillin, furfural and 5‐HMF) can be achieved increasing not only the productivity or respectively the current density, but also the selectivity towards EHD. For example, a similar approach was realized by Latsuzbaia et al. for the electrooxidation of 5‐HMF to FDCA realizing a pH neutral 0.75 M 5‐HMF feed concentration with a pH value adjustment to pH 12 with 10 M NaOH just before the reactor inlet.^[81]^ Industrial relevant current densities at low cell voltages should be achievable using a single pass flow reactor with porous 3D‐electrodes and high substrate concentration. Realization of such approaches to maximize current density and minimize the cell voltage should be focus of further studies. Also, as an extension of first promising studies,^[61]^ the use of crude feedstocks with impurities should be further investigated to enable an industrial interesting process. Experimental determination in a pilot scale demonstrator of essential key figures of merit, such as *STY* and *E*_s_, for the EHD products including workup strategies for product isolation are of paramount interest to quantify the capability of such processes for a subsequent scale‐up.^[82]^ Several scale‐ups of electroorganic processes have been recently realized and are covered within other reviews including reactor concepts and methodology.[^80^, ^83^]

## Summary and Outlook

The EHD of acrylnitrile (Monsanto process) is an example for an electrochemical synthesis at industrial scale based on fossil resources. The transformation of the chemical industry towards sustainability requires biobased feedstocks. Lignocellulose‐derived aromatic aldehydes, such as furfural, 5‐HMF and vanillin, are promising future platform chemicals for alternative value chains in the chemical industry.

In this review we have discussed the EHD of furfural, 5‐HMF and vanillin to their corresponding hydrodimers hydrofuroin, BHH and hydrovanilloin, which represent valuable building blocks for the synthesis of polymers, additives and fuels. The literature data on the EHD mechanism is reviewed and critically discussed. A high substrate concentration, high overpotentials of the cathode material for the competing HER, a low polarized cathode and alkaline media are critical reaction conditions when targeting EHD suppressing competing reactions such as hydrogen evolution or electrocatalytic hydrogenation. Green materials such as Zn or carbon‐based materials should be preferred over Pb, Hg and Cd.

Most of the studies reported in the literature were conducted in batch cells. Although first promising figures of merit, such as high FEs and yields, were obtained, a transfer from lab‐scale to technical process scale using electrochemical flow reactors is necessary in order to achieve industrial relevance. Therefore, in the next step work should be performed with respect to scale‐up of these interesting electrochemical reactions on flow reactor level. Furthermore, counter‐electrode electrochemistry should be reconsidered, since usually oxygen evolution at the anode was performed, which is neither thermodynamic nor economic beneficial. For example, the electrochemical oxidation of biobased feedstocks at the anode to valuable secondary products could lead to more favorable paired electrosyntheses.

## Conflict of Interests

The authors declare no conflict of interest.

## Biographical Information

*Robin Kunkel received his PhD in Electrochemical Engineering in 2022 from the Karlsruhe Institute of Technology (KIT, Germany). Currently, he is a Postdoctoral Researcher in the field of Electrosynthesis at the Fraunhofer Institute for Chemical Technology ICT (Germany). His research interest is mainly in electrochemical process investigation of biobased compounds and fine chemicals, electrochemical reactor design and electrocatalysis*.

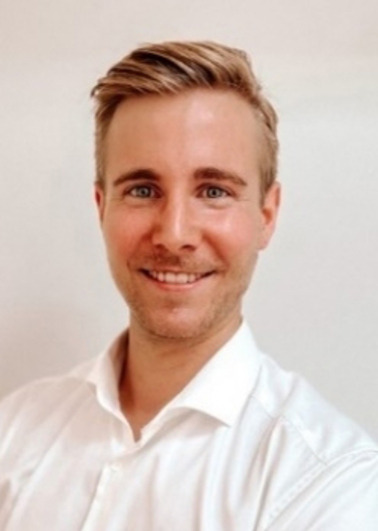

## Biographical Information

*Volkmar M. Schmidt studied chemistry at the University of Bonn (Germany) and received his PhD in 1991 from the University of Witten/Herdecke in the field of electrochemistry under the supervision of Prof. J. Heitbaum. After a postdoctoral stay on electrocatalysis in the group of Prof. W. Vielstich at the University of Bonn, he became project leader in the fuel cell program at the Jülich Research Centre with Prof. U. Stimming. Since 1997 he is professor at the Mannheim University of Applied Sciences working on electrocatalysis, fuel cells and electrochemical process engineering*.